# “*I Felt Safe*”: The Role of the Rapid Rehousing Program in Supporting the Security of Families Experiencing Homelessness in Salt Lake County, Utah

**DOI:** 10.3390/ijerph17134840

**Published:** 2020-07-05

**Authors:** Ivis García, Keuntae Kim

**Affiliations:** Department of City and Metropolitan Planning, University of Utah, 375 South 1530 East, Suite 235, Salt Lake City, UT 84112, USA; keuntae.kim@utah.edu

**Keywords:** homelessness, homeless shelter, subsidized housing programs, affordable housing, transitional housing, Homeless Management Information System (HMIS), Rapid Rehousing Program, Housing Urban Development, urban planning, poverty

## Abstract

Homelessness is a public health issue that many organizations are addressing through a Housing First Model. One such organization is The Road Home (TRH), which provides services to homeless individuals and families in Salt Lake County. TRH is perhaps best known for their emergency shelters, but the organization also administers the Rapid Rehousing Program (RRHP), designed to help families experiencing homelessness transition back into stable housing. Those experiencing homelessness tend to have high rates of chronic mental/physical disabilities as well as issues related to substance abuse. Having a home is the first step toward achieving some kind of stability in their lives. The RRHP allows families to find housing in the private rental market and will cover the initial costs and several months of rent for clients. While the program has been praised by policymakers and social service providers for helping homeless families find rental housing, there is no empirical research about participant perspectives regarding their residential (in)security. The research question of this article is: what is the role of the RRHP in supporting the security of families experiencing homelessness? Researchers collected qualitative data through focus groups and interviews with 31 participants, 23 families experiencing homelessness, two landlords, six case managers, and service providers. Lastly, we identify recommendations for program improvements based on information gathered from research participants. It is our hope that the information presented in this article can and will be used in a way that improves public health by increasing the residential security of families experiencing homelessness.

## 1. Introduction

The need for housing that is affordable to lower-income households has been steadily growing over the past several decades even before the Great Recession of 2007–2009, which devastated housing markets across the U.S. and the globe [1]. Factors that have contributed to the increased affordable housing demand since the 1980s in countries like the U.S., Australia, and Germany, to mention a few, primarily include: deindustrialization, changes in household income, and the lack of affordable housing production [2]. Furthermore, the increasing number of refugee and immigrant households over the last four decades in the U.S., Canada, and Europe accounts for a major share in the demand for rental housing [3]. Demand for rental housing is also increasing as baby boomers approach retirement and enter the affordable rental market. Household renters aged 45–64 make up one-third of the growth in renters in the United States [4].

Households unable to afford rent face an increasing risk of homelessness. Although this might come to the surprise of many, families make up about 40% of the U.S. homeless population [5]. It is estimated that about 25% of families that were homeless once will become homeless again in the near future due to their inability to reach economic security and, thus, residential security [6]. In the U.S., the levels of housing insecurity contribute to the following statistic: 1 out 10 people facing homelessness rely on family and friends for shelter [7]. There are other groups who experience high levels of housing insecurity as well. For example, 1 out of 10 veterans become homeless while 1 out of 11 formerly incarcerated are homeless and 1 out of 6 people leaving foster care are without a home [7]. According to the same source, *The State of Homelessness in America Report*, the odds of experiencing homelessness for the general population in the course of a year is about 1 out of 200, but, for those below the poverty line, is 1 out of 25 households.

Homelessness is a public health problem linked to other personal concerns for the homeless such as inadequate education, unemployment, and mental and physical health issues, which all contribute to residential insecurity [8]. Other social determinants of health such as the lack of access to food, healthcare, housing, etc. could be directly linked to the root causes of having poor health [9]. There are many physical and mental health comorbidities associated with homelessness such as trauma, diabetes, cellulitis, peripheral cardiovascular disease, hypertension, and respiratory diseases, to mention a few [10].

Furthermore, homelessness is also related to other social and community concerns such as declining public safety and property values along with an increased tax burden for social services and healthcare costs [11]. Americans agree that homelessness is a systemic and public health problem that society can aid in ameliorating. However, the methods to do so are not unilaterally agreed upon [12]. Salt Lake County, Utah, has progressively initiated a Federal U.S. Department of Housing and Urban Development (HUD) strategy to house the homeless as a more effective means of support than other transitional housing: The Rapid Rehousing Program (RRHP).

The RRHP typically has three major components: (1) recruiting landlords and finding appropriate housing, (2) providing moving and rent assistance, and (3) offering access to case management at home and other supportive services [13]. Some research suggests that RRHP is more cost effective than traditional transitional housing interventions and enables households to experience homelessness for shorter periods of time [14]. However, in general, there is not a lot of research addressing the role of the RRHP in supporting the security of families experiencing homelessness. This is the gap that this article seeks to fill [15]. Feeling secure is an important aspect of mental/physical health and overall quality of life [16]. From this perspective, housing a family experiencing homelessness could be seen as a public health intervention.

More specifically, the research question of this article is: what is the role of the RRHP in supporting the security of families experiencing homelessness? The aim of this study is to understand the relationship between security and housing among families who experience homelessness. This study analyzes qualitative data collected from focus groups, primarily with 23 homeless families/tenants who participated and qualified for the RRHP program. The research team also interviewed property owners and managers (2) as well as case managers and service providers (6) for a total of 31 participants. The partner for this research project was The Road Home (TRH), a non-profit service agency headquartered in Salt Lake County, Utah. The mission of TRH is “to help people step out of homelessness and back into our community” [17]. From 1 January 2015 to 31 December 2017 alone, TRH served 1462 homeless families, according to their Homeless Management Information System (HMIS) database [18]. TRH has administered the RRHP in Salt Lake County since its inception in 2009, and, since then, they have served over 3000 families [19].

The article is organized as follows. In Section 2, we present a literature review on homeless statistics and trends in the U.S., background on the RRHP, individual/structural factors affecting homelessness, perceptions of security among homeless families, and health and safety. Section 3 discusses the qualitative methods employed in this research. Our findings, in Section 4, gives insights into the subjective experiences of homeless families participating in the RRHP, whom overwhelmingly agree that the RRHP was key toward improving their family’s sense of security. Section 5, which involves the discussion, presents some highlights from the findings as well as future recommendations. Lastly, in the conclusion (Section 6), we identify recommendations for program improvements based on information gathered from families experiencing homelessness, landlords, case managers, and service providers.

Overall, all participants view TRH and the RRHP as a very useful, essential, and efficient tool in mitigating the affordable housing crisis in Salt Lake County for families experiencing homelessness. All would like the program not only to continue but also to expand since it currently serves only a small fraction of homeless families and it is time-limited. Participants also identified issues and concerns, and offered suggestions for improvement. It is our hope that the information presented in this article can and will be used in a way that improves residential security for families experiencing homelessness not only in Salt Lake County, but in other regions across the nation and even the globe.

## 2. Literature Review

Section 2 presents a summary of relevant U.S. federal government reports and scholarly work with the purpose of providing an overview of: (1) homeless statistics and trends in the United States, (2) the Rapid Rehousing Program, (3) the individual and structural factors affecting homelessness, (4) perceptions of security among homeless families, and (5) health and safety.

### 2.1. Homeless Statistics and Trends in the United States

Every year, on a single day in late January, a U.S. nationwide Point in Time (PIT) count of the homeless population is conducted by each Continuum of Care (CoC) within their service areas (city, county, state) and the count includes only those who meet the definition of literally homeless—“a person who lacks a fixed, regular, and adequate nighttime residence” [20]. It is important to note, however, that the McKinney-Vento Definition of Homeless is more expansive, and might include people doubling-up with family members [21]. In 2019, the most recent year for which PIT data is available, 567,715 homeless individuals were counted in the United States [22]. About 20% of those counted as homeless in 2019 had a severe mental illness while 16% suffered from chronic substance abuse and about half were living with a disability of some sort [23].

According to the national PIT, in any given night, 60,000 families in the U.S. are homeless. In Utah, there are about four families that are homeless out of every 10,000 families, which is below the national average. According to the National Alliance to End Homelessness, family homelessness has decreased substantially in the last decade [18]. For example, from 2007–2018, nationwide sheltered homelessness is estimated to have decreased by about 33,000 individuals [24]. The White House Council of Economic Advisors Report (2019) suggests this decrease may be artificial due to the fact that individuals in the streets, emergency shelters, and transitional housing are considered homeless while those in the RRHP are not [17]. According to the same White House report, if individuals in the RRHP were also counted as homeless, the number of homeless individuals would have increased by 66,000 people from 2007–2018 [17]. The next section introduces the reader to details about the RRHP and how its design might aim at achieving residential security among families experiencing homelessness.

### 2.2. The Rapid Rehousing Program

The Rapid Rehousing Program (RRHP) is a U.S. Department of Housing and Urban Development (HUD) Federal strategy used to quickly allow individuals and families experiencing homelessness to access (1) for a limited time, anywhere from one to 12 months, a rental unit in a scattered-site private apartment or home and (2) light-touch supportive services, which might include case management, medical services, and after school programs, to mention a few [25]. The program started 10 years ago as part of the American Recovery and Reinvestment Act of 2009 and it was enacted toward the tail end of the 2007–2009 Great Recession and Global Financial Crisis to help individuals and families gain self-sufficiency after a personal economic crisis [26]. The program spends 75% of the $15 billion that the U.S. Congress allocates annually, to house families alone, as opposed to single individuals, which might include veterans, people living with HIV, and physical or mental disabilities [27]. Families pay 30% of their income toward rent, which is the HUD standard for affordability [28].

The RRHP employs a “Housing First Model,” which aims at moving families out of the streets to the emergency shelter as soon as possible and placing them into either: (1) temporary housing (e.g., RRHP where the goal is rental housing in the community with home-based case management) or (2) permanent supportive housing, which is reserved for the chronically homeless who need case managers and other services on site [29]. The model, promoted originally by Dr. Tsemberis, prioritizes helping people experiencing homelessness to find and move in to housing first and then they can address the underlining issues that brought them into homelessness (e.g., finding employment, dealing with their substance abuse, etc.) through case management and other supportive wraparound services [30]. This model and other “high fidelity” Housing First programs, such as the Canadian national (formerly named) ‘At Home’ program, place emphasis on sustained and open-ended support [31]. The next section discusses the individual and structural factors affecting homelessness and, thus, creating insecurity.

### 2.3. Individual and Structural Factors Affecting Homelessness

Analyzing individual and structural factors are the primary tools to understand the reasons causing homelessness. According to Kim and García, individual factors are “demographic characteristics and personal physical characteristics associated with the risk of homelessness” [15]. Compounding factors such as age, gender, and race are important individual factors related to homelessness. In addition, mental and physical barriers are an essential individual characteristic to understand the risk of homelessness [32]. According to Bassuk and Rosenberg’ statement, “Many of their children had serious developmental and emotional problems. Homeless mothers had more frequently been abused as children…Psychiatric disabilities may have been another contributing factor” [8]. Family violence and the experiences of domestic abuse are not only the primary factors that cause mental issues among women, children, and youth, but also are the main reasons for entering homelessness [33]. Substance abuse is also associated with mental illness and involvement in the criminal justice system, which contributes to insecurity and homelessness [34].

Moreover, structural characteristics are major factors in homelessness. According to Kim and García, “Structural factors are associated with the risk of homelessness that measures social and economic conditions of homeless families before entering the shelter and becoming homeless” [15]. For example, these factors include employment status, income earnings, and cost of housing, to mention a few. In the U.S., rising housing costs are out of the most concern. About 37 million households in 2017 spent more than 30% and 18 million spent more than 50% [35]. Median housing costs, along with education and healthcare, continue to rise quicker than inflation and wages (even for two income households) in most major U.S. cities [36]. A study by Corinth (2017) found that, for every 1% increase in rent within a CoC, homelessness rates also increase by approximately 1% [37]. Housing prices in Utah have increased along with the proportion of households affected by not finding affordable housing and paying more than 30% toward rents or mortgages [38]. This study documents the experiences of homeless families that were given a chance to have a home again through the RRHP—even if in a temporary basis to get back on their feet, increase their levels of stability, and be able to afford housing on their own. The next section disuses what other studies have found about security among homeless individuals.

### 2.4. Perceptions of Security among Homeless Families

Lack of shelter and sleeping on the streets have associated with fewer personal safety and security. For example, one study that surveys 190 homeless individuals showed that more than half of them have been assaulted on the streets [39]. The statistics are higher among women who are more likely to be victimized such as being physically and sexually assaulted if they are unsheltered [40]. Unsheltered homeless have basically no privacy as they do everything in public. As Rose (2019) explains, “those facing unsheltered homelessness simply have no (private) space to retreat” to shower, eat, rest, and mend themselves [41]. In general, this line of research that compares shelter vs. unsheltered homeless individuals describes how those who are sheltered are more secure. They have warmth when it is cold, food, a place to sleep, medical assistance, and more. Popular media accounts, on the other hand, have a focus on how many unsheltered homeless actually avoid staying in shelters because they are hopeless, overcrowded, and full of drugs. Some have experienced personal assaults and robberies or they simply feel that they have to check their dignity at the door in exchange for that security [42].

Most studies, however, emphasize the idea of housing over shelters. According to the previously mentioned study of Kim and García (2019) about the RRHP in the Salt Lake City’s Road Home, the rental subsidy helped families from falling into homelessness again. Similarly, several studies from Australia have documented how, for homeless families, obtaining a subsidy to rent on their own also means to have some sense of residential security [43]. Another study of the Chicago-Low Income Housing Trust Fund, which provides rental assistance to homeless families, conducted focus groups with 49 tenants and found that, after receiving housing, homeless families experience positive changes such as (1) increased privacy (as one tenant put it “keys to your room”), increase amenities (private bath), decreased stress (“peace of mind” in the words of a participant), and more time with family members, including their children [44]. This study also found that the subsidy also contributes to economic security as predictable residence makes routine job commuting feasible and additional funds allow tenants to budget funds for travel, pay down credit card debt, buy clothing for children and grandchildren, and even budget funds to pay bills on time. Baron et al. (2011) found that, for the working tenants who lose their jobs or retire, the subsidy offered an opportunity to remain in the same apartment and adjust to the loss of income, which lowers the risk of homelessness. The final session of the literature review discusses public health concerns related to homelessness and safety.

### 2.5. Health and Safety

Homeless families commonly experience physical or mental disabilities. According to the 2015 Annual Homeless Assessment Report, 45% of those experiencing homelessness in the U.S. had some form of mental illness [45]. Besides trauma, people experiencing homelessness tend to suffer other chronic illnesses such as diabetes, cellulitis, peripheral cardiovascular disease, hypertension, respiratory diseases, and more [10]. Health conditions are much worse among the unsheltered populations [46]. In a previous study of TRH RRHP in Salt Lake City, authors found that 26.6% of heads of a household reported having mental health problems and 23.7% reported having chronic health problems [15].

Health issues among the homeless population are often exacerbated by their inability to access treatment, which is further worsened by unsanitary shelter and environmental exposures [47]. Without having a stable home, one experiencing chronic health issues, including substance abuse, also struggles to gain access to adequate care and to retain employment [48]. Homelessness is a public health issue that many organizations are addressing through a Housing First Model [30,31]. This model recognizes that having a home is the first step toward achieving some kind of stability and security.

Connell et al. (2014), who conducted semi-structured interviews with 19 adults who experience homelessness in the UK, discussed in detail some of the domains that affect quality of life once experiencing housing. These domains are related to reduced stress and mental health issues, in particular. This includes relationships and a sense of belonging, autonomy, hope, and hopelessness. In these interviews, people identified “feeling healthy, peaceful, calm, relaxed, stable, safe, and free” as important aspects of well-being and quality of life that improved their perceptions of overall health (p. 15).

Inside of the public health literature, more importance is being paid to the social determinants of health, which is a holistic approach to medicine, in where quality of life and well-being matter greatly [9]. Not being housed leaves individuals with limited choices and a diminished quality of life [16]. The premise of this article is that housing families experiencing homelessness could be considered a public health intervention. The next section presents details about the study participants and qualitative methods employed.

## 3. Methods

This study uses grounded theory, which means that researchers started with a basic research question: what is the role of the RRHP in supporting the security of families experiencing homelessness? [49]. The study utilized qualitative research methods to collect data regarding participants from three stakeholders related to the RRHP: (1) families experiencing homelessness who had participated from the RRHP program before, (2) current or former landlords leasing to program tenants, and (3) case managers (who advice families and connect them to resources) and social service providers (who provide services outside of TRH).

Most families were recruited at the emergency shelter. The entire family was invited and not only household heads. Most who attended were female (20 out of the 23), two men attended with their female partners, and one man was a single-father household. One lesbian couple also attended along with a grandmother/daughter household. This means that, in total, 19 households were represented in the sample. A demographic survey that participants completed showed that about half (52%) were in two-parent households. It was not clear if the partner was at the shelter or if these women were separated from their legal spouse or partner. On average, they had 1.83 children. The average age of participants was 37.5 years old. Most were white (69.5%) and some were Hispanic (26%). Only four out of the 23 people were employed. Two people were employed full-time and two were employed part-time earning between $7.25 and $11.00 an hour. About half had not finished high school or earned a GED and 40% reported having a disability. 

These families had received an RRHP apartment before, and they had returned to the homeless shelter after either being evicted or after not been able to pay for the apartment they had leased once the RRHP funding ceased. This was anywhere from 1 to 12 months. The families that participated in the focus groups at the shelter were approved for the RRHP for a second, third time, or even fourth time in at least one occasion to our knowledge. Case managers gave personal invitations to families they had pre-approved for the voucher and were waiting at the shelter to move into an RRHP funded apartment once again. Case managers emailed and called families that currently housed at an RRHP subsidized apartment, but only a few tenants came to those focus groups. Attendance to those focus groups was low, given that families were not already on-site. Free transit tokens were offered to families by case managers during scheduled social service meetings to facilitate their attendance. All tenants were offered $25 plus food for participating in the research project. Case managers, service providers, and landlords were recruited by sending invitation emails. Qualitative methods included focus groups and one-on-one interviews, conducted in October and November 2019. The focus groups were conducted with participants in the tenant and social service provider groups. Four focus groups took place with RRHP tenants, which totaled 23 participants. One focus group was conducted with social service providers totaling six participants. Two one-on-one interviews were conducted with landlord participants. Each of these meetings were approximately one-and-a-half hours long. A total of 31 people participated in the study. This study only used quotes from tenants, but the insights of other participants were employed in the overall analysis. The main topic of this article is the impact on shelter security of the tenant household (which is one of the six different topics addressed in the research). The engagement question for tenants was: How the rental assistance has impacted your life? There were a total of 10 exploration questions: (1) How your sense of security has changed?, (2) How would you describe your level of privacy and personal living space?, (3) How the subsidy helped you with your finances?, (4) How the subsidy helped you to meet your other needs?, (5) How your relationships with other household members have changed?, (6) How were your social connections after receiving the subsidy?, (7) Was the quality of life in your new neighborhood improved?, (8) After receiving the subsidy, did you try to find employment or educational opportunities?, (9) Did you seek other social services? and, (10) Lastly, what are your plans for the future? We also asked an exit question: Does anyone have any additional comments? The questions in the focus group protocol were open-ended and semi-structured, which means that researchers would not necessarily ask every single question, but they would ask follow-up questions based on the group interest but within the main topic [50].

A total of 18 researchers (principal investigator, research assistant, and students of a course titled Community Engagement in Planning) took the Institutional Review Board (IRB_00097742) exam and followed the IRB protocol approved by the University of Utah by one of the IRB administrators in 2017. Focus groups and interviews were audio-recorded, transcribed, and thematically coded using the Atlas.ti software program. A total of three researchers conducted each focus group. One leading facilitator asked the questions in the protocol. At the same time, the other two researchers aided the leading facilitator by collecting consent forms, audio recording, taking notes, welcoming participants, setting up the room and food, giving payments to participants, and obtaining their signatures for accounting purposes. The two assistants for the leading facilitator then divided the transcriptions as well as the coding in equal parts. Audio recordings were transcribed using Sonix, which is an automated transcription software, and then reviewed and edited for accuracy by researchers. In the end, the leading facilitator made sure the transcription was clean and that the coding was consistent. Before researchers coded the interviews, a workshop took place not only to learn Atlas.ti but to also practice the pre-assigned codes. A total of 20 selective codes were created by the principal investigator from a previous and very similar research project in homelessness as well as from the literature [51]. Triangulation was used to ensure that there was agreement among the researchers regarding what themes were present [52]. Students were instructed to think and write by thinking about vignettes or short stories that would demonstrate a particular theme [53]. A final presentation including a report was shared with the Road Home staff. This served as member checks with participants in the study regarding the results.

## 4. Results

The following is a snapshot of views and stories gathered from focus groups conducted with homeless families. All participants view the Road Home and the RRHP as a very useful, essential, and efficient tool in mitigating the affordable housing crisis in Salt Lake County for families experiencing homelessness. All would like the program not only to continue but also to expand since it currently serves only a small fraction of homeless families and it is time-limited. Participants also identified issues and concerns, and offered suggestions for improvement. 

Researchers identified four themes within the topic of (in)security: (1) (In)security in the emergency shelter, (2) kids’ behavior at the shelter vs. at home, (3) feeling at home, and (4) feelings of (in)security at the new home. Each topic is described below, with evidence employing quotes from families who participated from the RRHP.

### 4.1. (In)Security in the Emergency Shelter 

The following section describes how the sense of security among residents changed from the shelter to their new apartment. A participant shared: “The Road Home (operated shelter) has officers and a security, but I do not feel safe. People still bring some drugs. And some people suffer from mental problems. There is fighting, cursing each other out. This is just a rough place to be.” Some of our attendees asserted that the lack of private space and crowded shelter prevent them from feeling secure. A participant said:

*(At) the Road Home (operated shelter) we had none, like no privacy. You know, they’re walking around all the time, not just the staff, but like the other residents. They were stealing his son’s clothes and he didn’t keep his clothes in there, his shoes. They steal your food, like, it was terrible. It was like, there were bedbugs. That place is horrible. It was just like jail except for you could have your kid. Exactly the same feel. That’s what it reminded me of, like, I had a hard time there (prison). I hated being in there.*

A couple expressed being able to have better relationships when they were in an apartment, “You have better relationships with your kids and also with your partner too. As others said, in the Road Home (operated shelter) there is no privacy at all.”

For the most part, families reported having more security in the shelter than in the streets. They still experienced the lack of security, mostly coming from other residents at the shelter that have behavioral or mental health issues. As families expressed, the TRH does have security measures and policies to control robberies. Arms and drugs are not allowed in the shelter. Yet, some parents manage to break the rules, families reported. Families get clothes, food, medical healthcare, and everything they need, which increases their sense of security in a way. However, one of the challenges is that there is no privacy. Many stated that the shelter feels overcrowded with too many families sharing space and with 300 beds in total. Families described how their relationships degraded with the lack of privacy and contributed to their insecurity while at the emergency shelter.

### 4.2. Kids’ Behavior at the Shelter vs. at Home

Many participants had negative feelings about their situation and deep concerns about violence and how it impacts their kids’ physiological and mental health. They are also worried about the long-term impacts of homelessness in their kids’ behavior as well as their future. One participant believed that:

“*I mean, you can control who comes into your house and who doesn’t. At the shelter, you can’t control...Yeah. The other day, a 14-year-old grabbed my son by the neck and slammed him down on the ground for no reason...And I feel like the shelter just screws up the kid’s minds. Yeah. Well, it’s definitely not a place for kids.*”

Another participant talked about their kids’ behavior.

“*At the shelter, kids are very annoyed. Hum, they would get irritated for everything. I think this is unusual. This is their way of dealing with stress. They have temper outburst because this is not normal. So, I do worry about my kids’ mental health. I am concerned. I am not sure if this would have an impact long-term, so I want to have my own place again as soon as possible.*”

Another parent expressed:

“*It’s very hard to set up family rules in the shelter. Other kids do things differently because of their parents. And, then, your kids want to be able to do the same things that the other kids do, right? And, sometimes you get into disputes with the parents over your kids and their kids. This place has a culture and is not our family culture, but is the culture that the other parents and their kids create.*”

A mother said that she questioned if she was a good parent or not.

“*My children are my life. They keep me optimistic. Sometimes I worry because I am putting them through this. I sometimes do not believe on myself and then I question if I am a good parent. I love them, but I am not sure if, when they grow up, they would understand. Know this is difficult for them. I just hope they do not blame me.*”

Some people talked about how having a place made everyone in the family have their own space and get along. “*My boys were not fighting that much with each other or us when they had an apartment. They could go to their rooms and be grouchy there. We would be in the living room. Then they would come out and be calmer.*” Others talked about their children behaving better. “*They were not as angry when we had the subsidy. They were less frustrated. They slept better. Had their own space to sleep and play, do their homework, and concentrate.*”

Families experiencing homelessness had difficulties with other families staying at the shelter. Some families reported that other family’s behaviors affected their household. Furthermore, parents felt that, at the shelter, they could not make their own decisions and be independent. Families appreciated the level of security and independence that the RRH voucher provided them in comparison to the emergency shelter. We can see why case managers, social service providers, and even RRH guidelines advocate for moving families from the emergency shelter to their own rental housing as soon as possible. Thus, this would avoid trauma that could later result in mental health issues for children and their families.

### 4.3. Feeling at Home 

Another woman said that she could not really receive visitors at the Road Home operated shelter, but, when she had the subsidy, she could have family and friends over. This improved her social relations.

“*The benefits extended feelings about home and guests in our home. Sometimes, I have friends or my family. People felt welcome in my home. At the Road Home (operated shelter), it was very hard, people could not settle down, talk, or relax at their own leisure. I could give people some food and snacks. I liked welcoming people to my home. I felt more connected too.*”

Feeling at home was a topic brought to the discussion. A woman said,

“*When I moved to my apartment, I was moving on a budget. But I did not have to spend a lot in making my place feel like home. I received items from charity, or I save money by going to Goodwill and Dollar Tree. I felt amazing getting all that stuff. I got free food and stocked up my refrigerator. I did not feel homeless anymore.*”

Another woman expressed that she spent more money in the new apartment and that made her more financially insecure.

“*I did not figure out a budget. After moving out from the Road Home (operated shelter) to the apartment, I had some savings because I was receiving disability. So, I had an income, but I did not calculate how much I had for everything, my phone, rent, and food. I knew I already had expending problems. I knew. But I wanted to have a decent home. Instead of saving the money as backup for rent, I spent it in making the apartment nice. Buying furniture and things. It adds up!*”

As described above, having a place to call home really improves the relationships with family members as well as friends, which helps households to maintain or increase their social capital. Having a new home and a space to call your own is an “amazing” feeling, as one mother put it. For others, it was hard to make a budget. Families that have been living in poverty for many years struggle with money issues. Financial education, therefore, needed to help families to manage their money and make sure that they are able to create economic stability and maintain residential security, after they find employment and were able to afford a home on their own outside of the RRHP.

### 4.4. Feelings of (In)Security at the New Home

The attendees of the focus groups believed that, while the shelter is not a place for feeling safe, the RRHP had a positive impact on their life. This statement magnifies the role of assistance in their feeling of security, “When I had the Rapid Rehousing, I felt safe.”

While some of them believed that a private rental through the RRHP is safer than the shelter, being unsure about the length of assistance makes them feel insecure. For example, one of the attendees who is currently in the RRHP said:

“*I feel more secure knowing I’m not gonna lose my house or my apartment. I get nervous, though, every month because I don’t know if they’re going to help. Like, I’m not sure if they’re gonna keep helping me. So, I mean, they help, but then it’s kind of like stressful at the same time.*”

Several participants mentioned that not knowing for how many more months they would have the subsidy made them feel very insecure. Some people felt insecure because the place they ended up renting has its own problems. A woman expressed,

“*My housing case manager found me this place. It is the only place that would take me because I did time (went to jail or prison). But I have a family and other people do not. There is a lot of single men in the building and I do not feel that safe. There are guys shooting up in the hallways, sometimes they are passed out. I tell them ‘there are kids here!’*”

Another woman said,

“*I grew up in the Rose Park area. I had many apartments there too. I know how it is already. There is a lot of gang bangers. I know those guys. I did not want to end up there again. But I did.*”

A gentleman expressed,

“*I know the complex that you are talking about in Rose Park. There is a lot of drug dealing going on there. Officers go there all the time. They ask for information. A lot of people who have felonies, like me, end up there. Everyone concentrates in the same housing developments. The people are trouble, and that affects our families, but they would not take us anywhere else.*”

Some long-term tenants mentioned that they would like to move because of bad influences, feeling insecure in their neighborhood, bad landlords, jobs, school, etc., but they cannot because they have criminal records, felonies, and other barriers that makes it very hard for them to find quality housing.

## 5. Discussion

Housing security has been central to HUD’s goal as established by its mission “to create strong, sustainable, inclusive communities and quality affordable homes for all. HUD is working to strengthen the housing market to bolster the economy and protect consumers, meet the need for quality affordable rental homes, utilize housing as a platform for improving quality of life, and build inclusive and sustainable communities free from discrimination” [54]. Within this goal in mind, HUD calls for the use of housing resources to improve quality of life. Public health literature has emphasized the importance of the social determinants of health including a number of measures of quality of life and well-being [9]. The Housing First model has supported the idea that, when people are housed, they can address the underlying issues, including substance abuse, physical issues, and health issues [26,27,28].

Connell et al. (2014) identified “feeling healthy, peaceful, calm, relaxed, stable, safe, and free” as key aspects for improving quality of life and overall health among individuals experiencing homelessness (p. 15). This study also discussed the importance of improving relationships, having a sense of belonging, autonomy, and having hope for the future as key domains of quality of life. Similarly, this research has found that the RRHP does contribute to improving family’s quality of life by procuring for families experiencing homelessness and residential insecurity. Along similar lines, the RRHP seems to enable TRH and other social service providers to better leverage their efforts. Securing affordable housing for clients, who are homeless, increases the potential to improve client security, autonomy, and stability.

As described above, the RRH voucher helps clients to feel more secure by helping them move out of the emergency shelter. The focus group showed that some tenants did not feel safe in the shelter. Others reported feeling that they did not have privacy and TRH felt overcrowded. There were complaints about other residents stealing from them or abusing their children. TRH residents also reported that their kids misbehave in the shelter and that they learn new behaviors from other children, which undermine their parenting. Parents felt that, in the shelter, it was harder to discipline their children and some felt that the mere fact of being in the shelter could be perceived as a sign of them being bad parents or increasing their kids’ likelihood of developing mental health issues.

Once families had a place on their own and autonomy, they felt safer and more secure. Their children behave better after having their own space. Kids were able to do their homework and concentrate. Parents felt they had better relationships with their children and their spouses after being housed. Some felt that they could have closer relationships with friends and family because they could host people when they had an apartment. People expressed that, having a place to call home and having a sense of belonging, was very important to them. Many reported having hope for the future.

For some, insecurity continued, even after being housed. For example, a few expressed that moving into a new place, contributed to more expenses and many did not know how to budget. Others felt less secure in the new housing because of the incidence of crime in their building. Additionally, some expressed concern about what would happen once their subsidy ended. This means that, although they felt they had residential security, they feared becoming housing insecure once the subsidy ended. Not knowing for how long they would have the assistance made them feel insecure. A recommendation would be for TRH to lay out more clear expectations for families about how long they would have the RRH subsidy, so they could plan ahead. More intensive case management, but, particularly, financial education could help families to increase their housing security in the long run.

## 6. Conclusions

This study is the first one trying to understand security among homeless families that move from the shelter to a rental on their own. Yet, even when it is the first study looking at homeless families being housed specifically, other researchers such as Rodriguez (2009) et al. and Nyamathi et al. (2000) have discussed how homeless individuals, especially women, seek emergency shelters as a way of improving their security, compared to living on the streets. The Connell et al. (2014) study showed that people with mental health issues, whom also struggle with homelessness, had feelings of relief and felt safer once they were housed. Having a home gave them a sense of having control and autonomy, which increased their “self-functioning.”

Most of the research on homelessness has created a hierarchy between staying in a shelter and having an actual home or your own “keys” to use the words of those with lived experience of homelessness [36,37,38,39,40,41]. This is not a surprise. Society at large would agree that having a home is better than staying in an emergency shelter. The Housing First model (e.g., Tsemberis et al.) has discussed why having a place to call home is simply better. A large part of the argument is that it allows individuals to address the underlying issues that brought them into homelessness to begin with, whether it is unemployment, mental illness, substance abuse, or something else [26,27,28]. This study is a contribution toward understanding why having a home is better. Essentially, personal safety and security is improved. Although improved security and safety was expected, this qualitative research has also described the how.

This statement magnifies the role of the RRHP in creating some level of residential security for families who experienced homelessness. “When I had the Rapid Rehousing, I felt safe.” Overall, TRH and social service providers appreciate the RRHP as it provides their clients with security and consistency in their lives, and allows them to offer a more light-touch and on-a-needed basis social services. If clients are housed, it is easier for them to excise self-care and take their pills. If their clients are housed, it is easier to communicate with them, call them at their home, and drop-by. Clients in stable housing are also more likely to make appointments with the case workers, doctors, and health care providers. Housing allows many of their clients to reconnect with family members. Children can visit their parents, grandchildren can visit their grandparents, parents can support kids and young adults, and so on. Quality of life is completely interlinked with having a place to call home [16,54]. This is why being house is one of the social determinants of health [9].

As with all programs that assist very low-income people, resources and capacity is often an ongoing struggle. In addition, with the limited number of RRH vouchers, TRH has been stretched and really challenged to find new sources of funding and new ways of doing more for less. Expanding the reach of its programs raises a challenge for the RRHP since it would have to maintain its current funding base and also increase its resources over time. While expansion may be a daunting proposition given the current funding environment, the need is clearly evident, as documented in this article. If TRH were to expand its resources and offer the RRHP to home households for a longer period of time, it would be in a position to create housing security and improve public health throughout the region. Due to the infrastructure already in place and the experience TRH has accumulated over the years, the transaction and administrative costs associated with any expansion would be minimal, which allows most of the new resources to be channeled to meet the needs of the most vulnerable residents of Salt Lake County so they can feel safe.
